# Fibroblast p90RSK induces epithelial transdifferentiation through oxidative stress‐mediated β‐catenin pathway

**DOI:** 10.1002/ctm2.1128

**Published:** 2023-01-08

**Authors:** Chaowen Shi, Ling Lin, Kebin Hu

**Affiliations:** ^1^ Division of Nephrology Department of Medicine Penn State University College of Medicine Hershey Pennsylvania USA; ^2^ Department of Cellular and Molecular Physiology Penn State University College of Medicine Hershey Pennsylvania USA

Dear Editor,

Chronic kidney disease (CKD), with interstitial fibrosis as the prominent hallmark, is one of the most common chronic diseases in the world. Interstitial fibroblasts and tubular epithelial cells, as well as crosstalk of each other, play a fundamental role in CKD pathogenesis and progression. Here, we defined the role and signaling mechanism of p90RSK (RPS6KA1), the main isoform of RSKs in kidney,^1^, ^2^, ^3^ which is activated during CKD, in fibroblast‐mediated epithelial transdifferentiation and CKD pathogenesis.

Our recent work has shown that p90RSK is activated, primarily in fibroblast‐specific protein‐1 (FSP‐1)‐positive fibroblasts, during CKD, and its activation closely correlates to the extent of kidney fibrosis.^4^ We have generated an FSP‐1‐specific p90RSK transgenic mouse (RSK‐Tg) by cross‐breeding p90RSK‐Tg^flox^ mouse and S100A4 (FSP‐1)‐Cre mouse (The Jackson Laboratory). Although other types of cells, such as macrophages,^5^ also express FSP‐1, it has been reported in an EGFP reporter mouse that FSP‐1 promoter only drives Cre recombinase expression in renal interstitial fibroblasts.^6^ As we previously reported,^4^ RSK‐Tg mice and their wildtype counterparts (RSK‐wt) show similar phenotypes. However, after obstructive injury, RSK‐Tg mice displayed exaggerated tubular epithelial damage and interstitial fibrosis as demonstrated by periodic acid‐schiff (PAS) staining (Figure 1A), renal total collagen content (Figure 1B) and Western blot of FSP‐1 (Figure 1C,D), compared to RSK‐wt mice. Normal kidney structure and functions rely on epithelial integrity and their interactions with other cells. Epithelial transdifferentiation, that is, epithelial‐mesenchymal transition (EMT), is a reversible process involving loss of epithelial integrity and gain of contractility and mobility and contributing to CKD. In contrast to RSK‐wt mice, renal histology of RSK‐Tg mice displayed dramatically increased dilated tubules (Figure 1A, indicated by *), suggesting more severe epithelial integrity loss after obstructive injury. RSK‐Tg mice also had significantly higher level of matrix metalloproteinase‐9 (MMP‐9) (Figure 1E,F) and remarkably lower level of Ecadherin (Figure 1G,H) than RSK‐wt mice. Additionally, RSK‐Tg mice showed dramatical induction of αSMA (Figure 1I,J) in the obstructed kidneys, indicating an active EMT process. Double immunofluorescence staining further confirmed that these injured lectin‐positive epithelial cells (red) also de novo expressed mesenchymal marker of FSP‐1 (green, Figure 1K,L). We established a novel fibroblast‐epithelial co‐culture system using primary renal fibroblasts from RSK‐Tg and RSK‐wt mice, as well as HKC‐8 epithelial cells (Figure 1M). Consistent with our in vivo findings, RSK‐Tg fibroblasts markedly induced epithelial transdifferentiation as indicated by suppression of Ecadherin (Figure 1N,O) and induction of αSMA (Figure 1P,Q), vimentin (Figure 1R,S) and fibronectin (Figure 1T,U), as well as enhanced mobility (Figure S1). Thus, fibroblast p90RSK not only impairs tubular epithelial integrity but also induces epithelial transdifferentiation.

**FIGURE 1:**
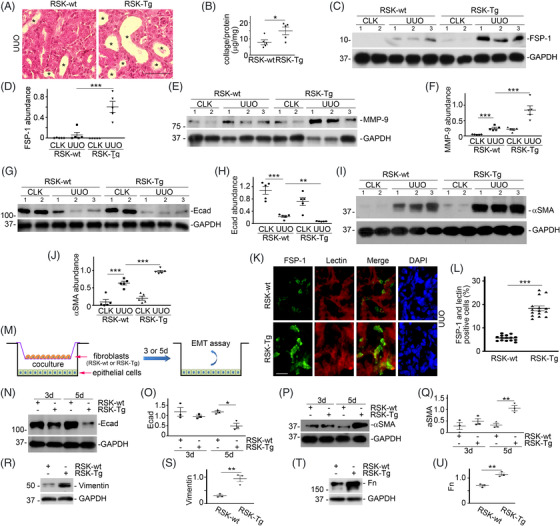
Fibroblast‐specific p90RSK exaggerates obstruction‐induced renal fibrosis and promotes epithelial transdifferentiation. Fibroblast‐specific p90RSK transgenic mice (RSK‐Tg) and their littermate controls (RSK‐wt) were subjected to UUO for 7 days. (A) PAS staining of kidney sections, 400×, bar: 50 μm. * indicates the dilated tubules. (B) Renal total college content quantitation. *p < .05, *n* = 5 mice/group. Western blot for fibroblast‐specific protein‐1 (FSP‐1) and glyceraldehyde‐3‐phosphate dehydrogenase (GAPDH) (C), MMP‐9/GAPDH (E), Ecadherin (Ecad)/GAPDH (G) and αSMA/GAPDH (I). Relative abundance of FSP‐1 (D) MMP‐9 (F), Ecad (H) and αSMA (J) **p < .01, ***p < .001, *n* = 5 mice/group. Number indicates individual mouse. (K) Double immunofluorescence staining of FSP‐1 (green) and Lectin (proximal tubular epithelial marker, red), bar: 20 μm. (L) Quantitation of both FSP‐1 and Lectin‐positive cells. ***p < .001, *n* = 5 mice/group. (M) Illustration of primary fibroblasts (RSK‐Tg and RSK‐wt) and epithelial cells (HKC‐8) co‐culture. After serum starvation overnight, fibroblasts were co‐cultured with HKC‐8 cells for additional 3 and/or 5 days. Then, HKC‐8 cells were harvested and subjected to Western blot for Ecad (N), αSMA (P), vimentin (R) and fibronectin (Fn, T), as well as respective GAPDH. Quantitation of abundance of Ecad (O), αSMA (Q), vimentin (S) and Fn (U). *p < .05, **p < .01, *n* = 3 experiments. CLK, control kidney; EMT, epithelial‐mesenchymal transition; UUO, unilateral ureter obstruction

Immune staining of 4‐hydroxynonenal (4‐HNE), a specific marker of reactive oxygen species (ROS), was used to examine ROS level in the fibrotic kidneys from RSK‐Tg and RSK‐wt mice. RSK‐Tg mice showed enhanced 4‐HNE expression predominantly in epithelial cells compared to RSK‐wt mice (Figure 2A). 4‐HNE staining concentrated near the basal compartment of tubules suggesting a source of oxidative stress from the interstitium surrounding epithelium. These RSK‐Tg fibroblasts consistently produced significantly higher level of H_2_O_2_ in medium for up to 5 days after co‐culture with epithelial cells (Figure 2B). However, YCG063, a specific ROS inhibitor,^7^ almost eliminated RKS‐Tg fibroblast‐induced EMT as indicated by restoration of Ecadherin (Figure 2C,D) and suppression of αSMA by Western blot (Figure 2E,F). Notably, RSK‐Tg mice displayed dramatical induction of β‐catenin in both cytosol and nuclei of epithelial cells in obstructive kidneys (Figure 2G). We further uncovered that RSK‐Tg fibroblasts induced β‐catenin epithelial accumulation (Figure 2H,I) and its nuclear translocation (Figure 2J) after co‐culture. However, siRNA knockdown of epithelial β‐catenin abolished RSK‐Tg fibroblast‐mediated EMT as demonstrated by restoration of Ecadherin (Figure 2K,L) and suppression of αSMA by Western blot (Figure 2 M,N). Efficiency of siRNA knockdown was also validated by another set of β‐catenin siRNA (siRNA1, Figure S2D–F). Thus, we concluded that oxidative stress‐induced β‐catenin mediates fibroblast p90RSK‐triggered EMT.

**FIGURE 2:**
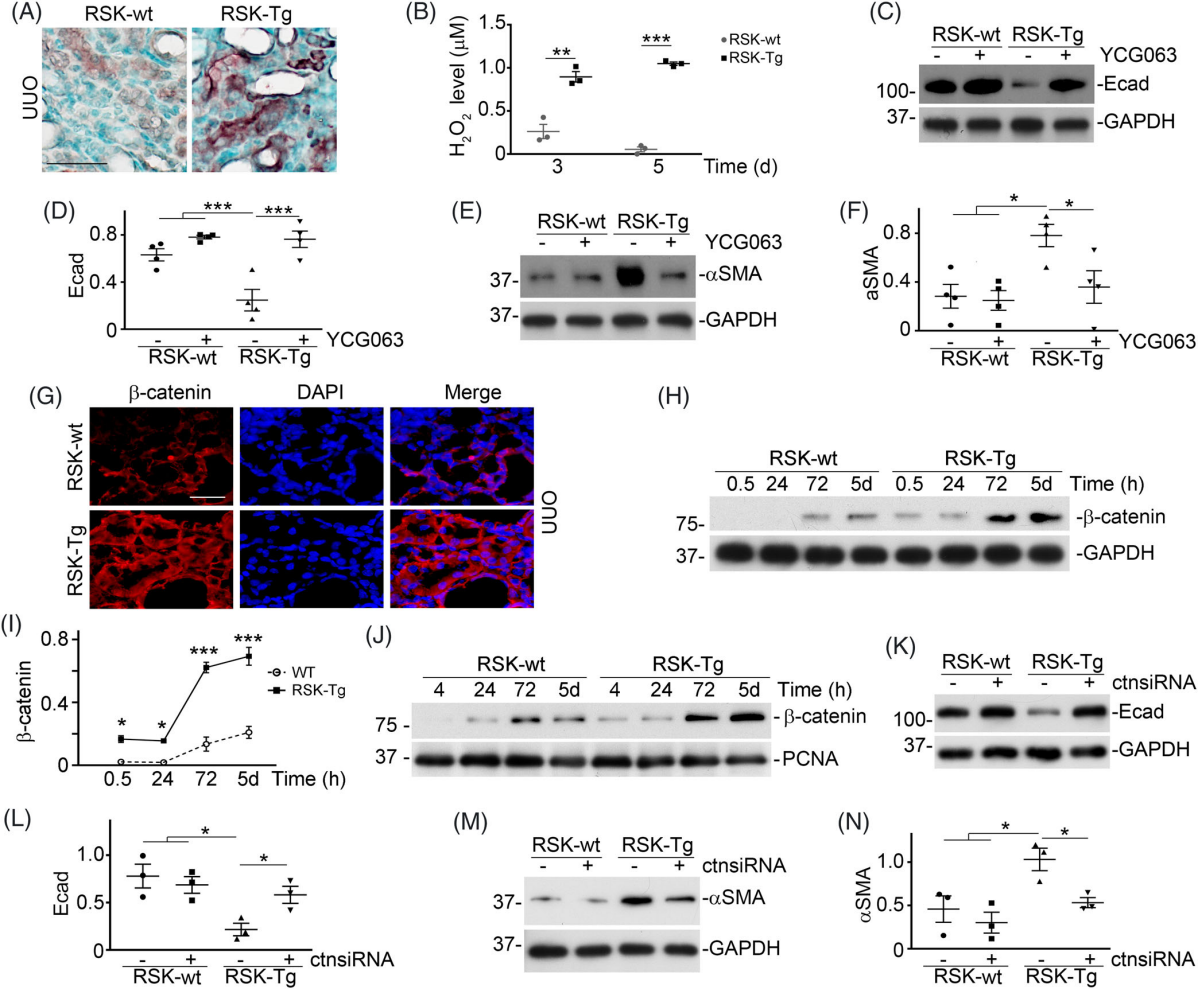
Reactive oxygen species (ROS)‐induced β‐catenin mediates fibroblast‐specific p90RSK‐induced tubular epithelial transdifferentiation. (A) Obstructed kidneys were subjected to immune staining for 4‐HNE, 400×, bar: 50 μm. Primary fibroblasts from fibroblast‐specific p90RSK transgenic mice (RSK‐Tg) or littermates (RSK‐wt) were in co‐culture with HKC‐8 cells for 3 or 5 days. Then, H_2_O_2_ level in the co‐culture medium was measured (B), **p < .01, ***p < .001, *n* = 3 experiments. Additionally, 50 nM YCG063 was added into the co‐culture for 5 days, followed by Western blot for Ecad/GAPDH (C) and αSMA/GAPDH (E) in HKC‐8 lysates. Quantitation of Ecad (D) and αSMA (F) abundance, *p < .05, ***p < .001, *n* = 3 experiments. (G) Obstructed kidneys were subjected to immune staining for β‐catenin, 400×, bar: 20 μm. After co‐culture with primary RSK‐Tg and RSK‐wt fibroblasts for various period as indicated, HKC‐8 cells were harvested for Western blot of β‐catenin and GAPDH (H). (I) Quantitation of β‐catenin abundance. *p < .05, ***p < .001, RSK‐Tg versus RSK‐wt, *n* = 3 experiments. (J) Western blot of β‐catenin and proliferating cell nuclear antigen (PCNA) in HKC‐8 nuclear extracts. HKC‐8 cells were transfected with control or β‐catenin siRNAs, followed by co‐culture with RSK‐Tg or RSK‐wt fibroblasts for 5 days. HKC‐8 lysates were subjected to Western blot for Ecad/GAPDH (K) and αSMA/GAPDH (M). Relative abundance of Ecad (L) and αSMA (N) in epithelial lysates, *p < .05, *n* = 3 experiments. ctnsiRNA, β‐catenin siRNA; UUO, unilateral ureter obstruction.

In summary, this study defined that p90RSK‐overexpressing fibroblasts produce excessive H_2_O_2_, causing ROS and β‐catenin accumulation in the surrounding epithelium. β‐catenin triggers aberrant epithelial transdifferentiation through inducing T cell factor/lymphoid enhancer factor (TCF/LEF)‐mediated gene expression. These myofibroblasts via EMT process, in turn, produce excessive matrix resulting in fibrosis (Figure 3). Excessive ROS not only induces epithelial β‐catenin accumulation and nuclear translocation^4^ (Figure S2A) through oxidation of cysteine‐205 and ‐208 residues of nucleoredoxin, which, in turn, dissociates from Dishevelled and stabilizes β‐catenin^8^ causing subsequent epithelial injuries (Figure S2B,C) through LEF‐1 pathway (Figure S2G–K) but also further activates p90RSK,^9^, ^10^ forming a vicious cycle of amplification. These results, in combination with our previous finding that fibroblast p90RSK promotes epithelial apoptosis, a histological characteristic of acute kidney injury, through oxidative stress‐mediated β‐catenin/FOXO1 pathway,^4^ illuminate the pivotal role of β‐catenin in p90RSK‐induced fibroblast‐epithelial communication. It is presumable that β‐catenin acts as a control knob to induce epithelial apoptosis causing disruption of kidney structure and/or trigger epithelial transdifferentiation resulting in fibrotic scars by pivoting to its respective downstream transcription factor FOXO1 or TCF/LET.

**FIGURE 3:**
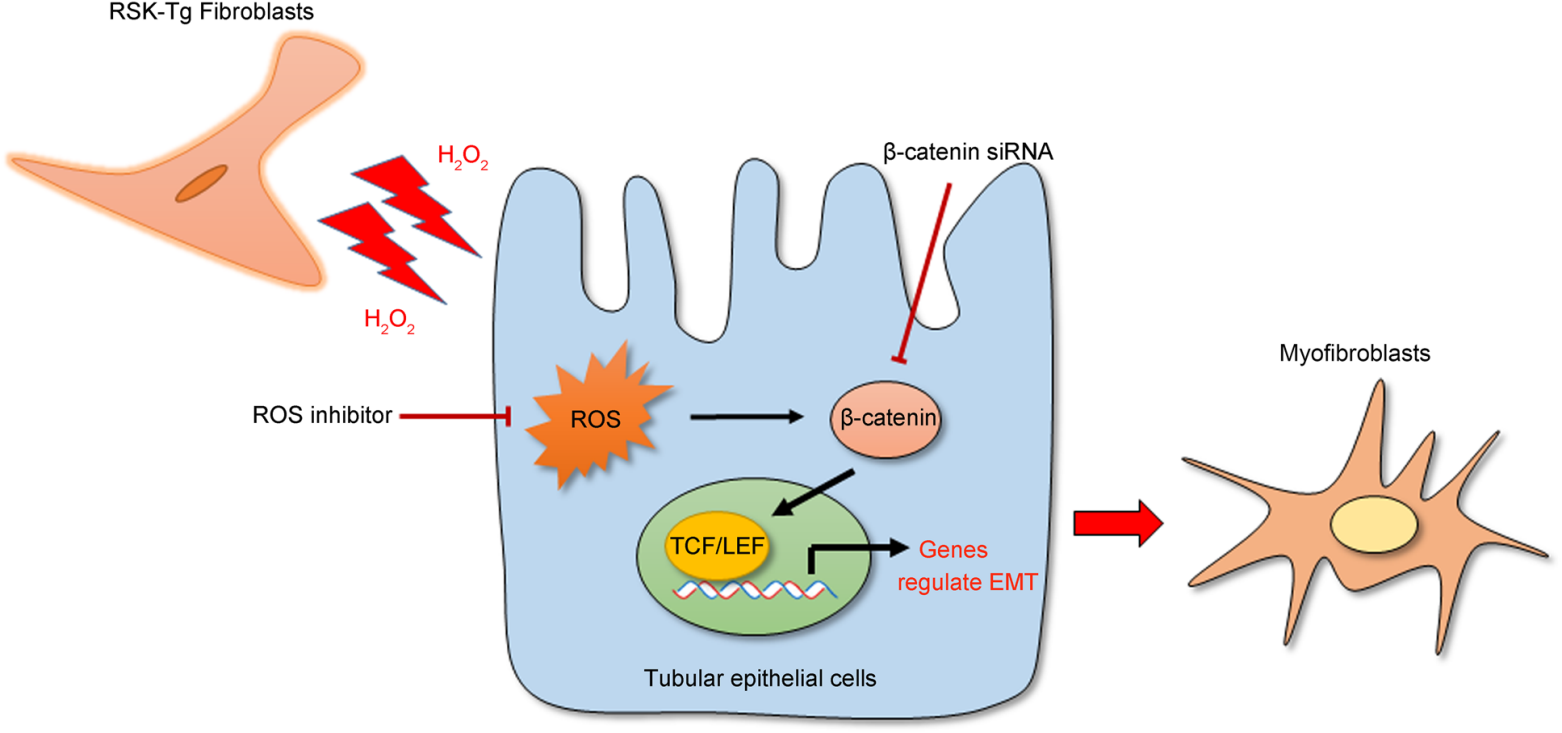
Schematic illustration of fibroblast p90RSK‐induced epithelial transdifferentiation. p90RSK‐transgenic fibroblasts surrounding the tubular epithelial cells generate excessive H_2_O_2_, causing epithelial accumulation of reactive oxygen species (ROS), which, in turn, induces cytosol accumulation of β‐catenin, followed by activation of TCF/LEF to initiate epithelial transdifferentiation, that is, epithelial‐mesenchymal transition (EMT) process, expanding the population of interstitial myofibroblasts and eventually leading to renal fibrogenesis.

It is clear that p90RSK induces fibroblast‐mediated epithelial transdifferentiation through a novel signaling mechanism involving ROS and β‐catenin.

## CONFLICT OF INTEREST

The authors declare that there is no conflict of interest that could be perceived as prejudicing the impartiality of the research reported.

## Supporting information

Supporting Information

Supporting Information

Supporting Information
