# Supplementary material for: Fibroblast p90RSK induces epithelial transdifferentiation through oxidative stress‐mediated β‐catenin pathway
Source: Clin Transl Med. 2023 Jan 8;13(1):e1128. doi: 10.1002/ctm2.1128 (PMC9826782; doi:10.1002/ctm2.1128)
Supplement: Supplementary file 1 — Supporting Information [file CTM2-13-e1128-s003.docx]

**Figure S1. p90RSK fibroblast-mediated transdifferentiated epithelial cells acquires migratory ability.** Primary RSK-Tg and RSK-wt fibroblasts were in coculture with HKC-8 cells for 30 hours or 5 days followed by transwell migration assay (A) and wound healing assay respectively (C), bar: 200 µm. (B) Quantitation of migrated cells, ****P*<0.001, n=3 experiments. (D) Quantitation of wound healing, ****P*<0.001, n=3 experiments.

**Figure S2. H_2_O_2_ induces epithelial nuclear translocation of β-catenin, which mediates fibroblast p90RSK-induced EMT.** (A) HKC-8 cells were treated with 1.5 µM H_2_O_2_ for 1 hour, followed by immunofluorescence of β-catenin (green) and phalloidin (red), bar: 25 µm. HKC-8 cells were incubated with H_2_O_2_ at indicated concentrations for 4 days, followed by Western blot for Ecad and GAPDH (B) and αSMA (C). (D) HKC-8 cells were transfected with control or two sets of β-catenin siRNA (ctnsiRNA and ctnsiRNA1), followed by coculture with RSK-Tg or RSK-wt fibroblasts for 5 days. HKC-8 lysates were subjected to Western blot for β-catenin (D), Ecad/GAPDH (E) and αSMA/GAPDH (F). HKC-8 cells were transfected with control and LEF-1 siRNAs (40 and 80 nM), followed by incubation with 25 or 50 µM H_2_O_2_ for 4 days. HKC-8 lysates were subjected to Western blot for LEF-1 (G), Ecad/GAPDH (H) and αSMA/GAPDH (I). (J) Relative abundance of Ecad, **P*<0.05, ***P*<0.01, n=3. (K) Relative abundance of αSMA, **P*<0.05, ***P*<0.01, n=3. ctnsiRNA: β-catenin siRNA.
