# Supplementary figures and images for: Fibroblast p90RSK induces epithelial transdifferentiation through oxidative stress‐mediated β‐catenin pathway
Source: Clin Transl Med. 2023 Jan 8;13(1):e1128. doi: 10.1002/ctm2.1128 (PMC9826782; doi:10.1002/ctm2.1128)

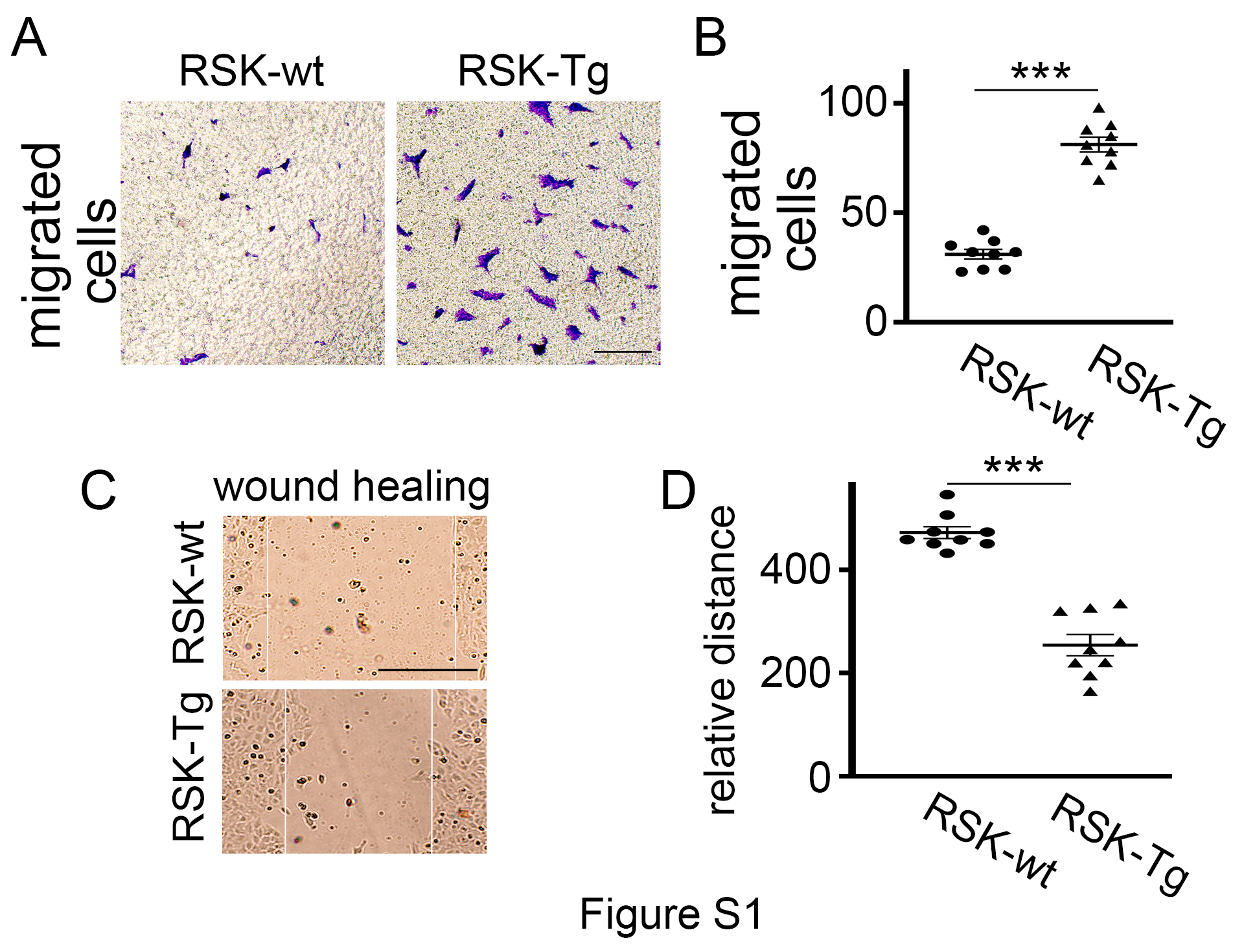

Supplement: Supplementary file 2 — Supporting Information [file CTM2-13-e1128-s001.tif]

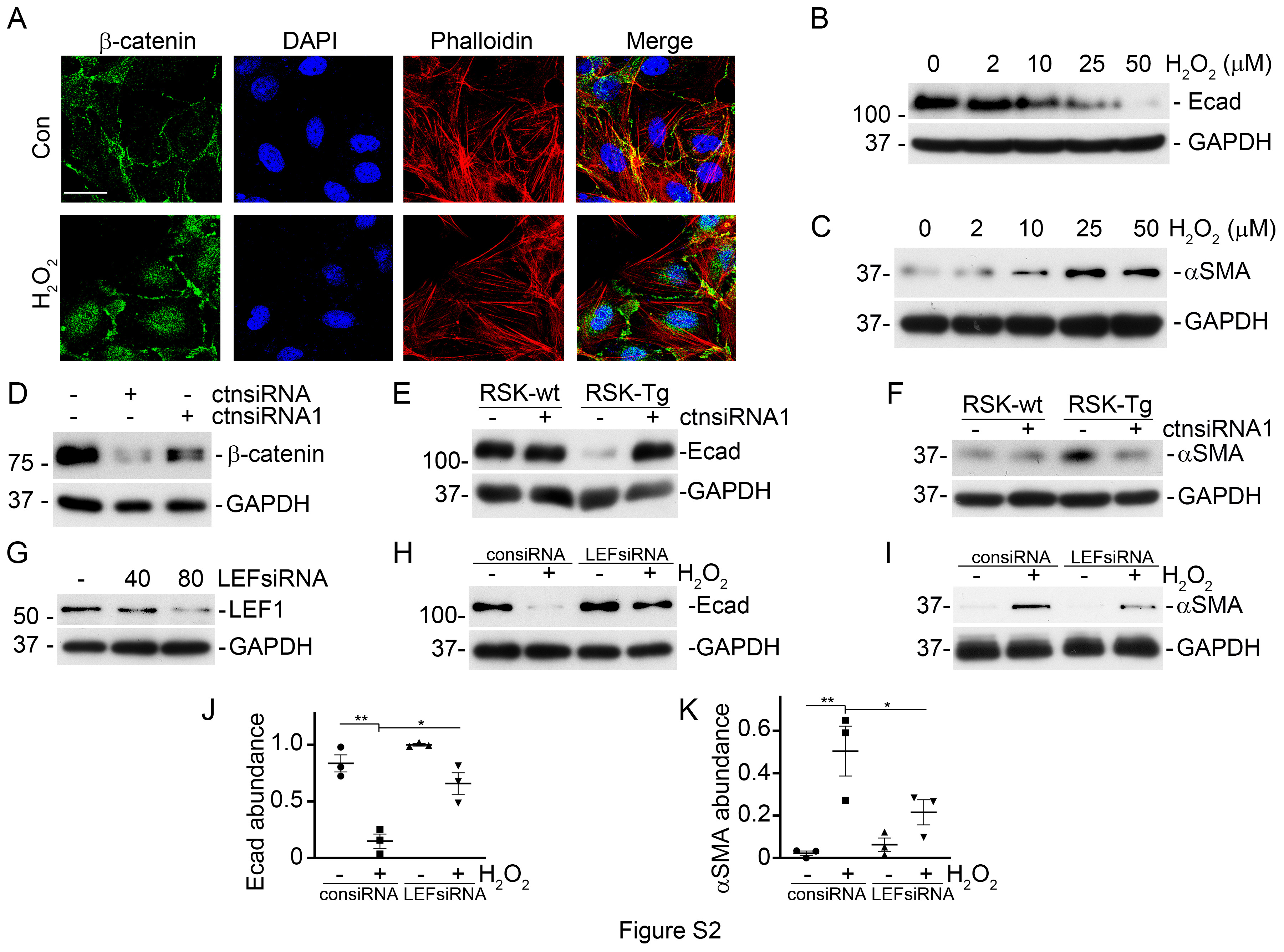

Supplement: Supplementary file 3 — Supporting Information [file CTM2-13-e1128-s002.tif]
